# Must Clinics Replace 2D by 3D Environments for an Efficient Training of Laparoscopic Novices? A Critical Analysis of the Learning Curve for Basic Skills

**DOI:** 10.3389/fsurg.2021.792107

**Published:** 2022-01-17

**Authors:** Maik Sahm, Clara Danzer, Alexis Leonhard Grimm, Christian Herrmann, Rene Mantke

**Affiliations:** ^1^Department of Surgery, Brandenburg Medical School, University Hospital, Brandenburg, Germany; ^2^Department of Surgery, DRK Kliniken Berlin Köpenick, Berlin, Germany

**Keywords:** learning curve, 2D laparoscopy, 3D laparoscopy, surgical skill, novice

## Abstract

**Background and Aims:**

Published studies repeatedly demonstrate an advantage of three-dimensional (3D) laparoscopic surgery over two-dimensional (2D) systems but with quite heterogeneous results. This raises the question whether clinics must replace 2D technologies to ensure effective training of future surgeons.

**Methods:**

We recruited 45 students with no experience in laparoscopic surgery and comparable characteristics in terms of vision and frequency of video game usage. The students were randomly allocated to 3D (*n* = 23) or 2D (*n* = 22) groups and performed 10 runs of a laparoscopic “peg transfer” task in the Luebeck Toolbox. A repeated-measures ANOVA for operation times and a generalized linear mixed model for error rates were calculated. The main effects of laparoscopic condition and run, as well as the interaction term between the two, were examined.

**Results:**

No statistically significant differences in operation times and error rates were observed between 2D and 3D groups (*p* = 0.10 and *p* = 0.72, respectively). The learning curve showed a significant reduction in operation time and error rates (both *p*'s < 0.001). No significant interactions between group and run were detected (operation time: *p* = 0.342, error rates: *p* = 0.83). With respect to both endpoints studied, the learning curves reached their plateau at the 7th run.

**Conclusion:**

The result of our study with laparoscopic novices revealed no significant difference between 2D and 3D technology with respect to performance time and the error rate in a simple standardized test. In the future, surgeons may thus still be trained in both techniques.

## Introduction

Laparoscopy is a state-of-the-art technique in abdominal surgery clinics today due to the undeniable benefits of its lower invasiveness. A reliable recording of the intraoperative site is essential for successful laparoscopy, and the technical equipment has accordingly improved constantly. Already, the development of high-definition (HD) camera systems with higher resolution, more brightness, and less distortion resulted in measurable technological progress of 2D (two-dimensional) video systems in practice (1). 2D environments were, for a long time, the method of choice, before 3D (three-dimensional) components successively became more established in routine surgery. The first stereoscopic 3D devices were developed in the 1990s, providing a different spatial view of the operation field with improved outcomes for the patient, while the introduction of 4K monitors for 2D laparoscopy with 4-fold higher resolution as compared to 2D/HD led to a further improvement of the monoscopic view. Today, high-resolution 2D or 3D video systems are an integral part of, basically, all modern operating theaters, and clinics currently often use both in parallel.

Experienced surgeons often prefer monoscopic special features to gain a three-dimensional impression despite the lacking stereoscopic view in 2D systems (2), especially if they experience side effects like eye strain, vertigo, or discomfort under 3D vision technologies (3). A stereoscopic view might, nevertheless, be beneficial due to an improved depth perception, and many studies, indeed, demonstrate advantages of 3D over 2D/HD systems, which are reflected in a reduced performance time and lower number of errors in daily clinical practice.

Comparative studies of 2D and 3D laparoscopy already date back to the 1990s (4). Buess et al. showed 1996 an error reduction of 43% and a 32% reduced performance time under 3D as compared to a 2D view (5). While the benefits of 3D environments in practice are evident, the question remains if a costly technical change from 2D to 3D is really required to improve the acquisition of basic laparoscopic skills in a standardized training setting. If the superiority of 3D systems is demonstrable in the learning curve of inexperienced medical students, a direct entry into 3D laparoscopy should be recommended to enable faster integration into a clinical daily routine.

## Materials and Methods

### Sample Size Calculation and Endpoints

In a pilot study using the 2D technique, test persons (*n* = 3) started on average with 224 s in Experiment 1 and ended, on average, at 152 s in Experiment 10.

We defined a 15% reduction in time (23 s) for 3D compared to 2D technique as a meaningful improvement. With an estimated standard deviation of 26 s in the pilot study, a group size of 22 test persons per group is required to achieve a power of 80% by assuming the usual alpha-level of 5%.

For this prospective, randomized controlled study, 45 laparoscopic novices were recruited. All the participants were students of the Brandenburg Medical School Theodor Fontane, the Brandenburg University of Applied Sciences, or other training facilities.

All the participants were surveyed in a questionnaire with respect to gender, wearing of glasses, video gaming frequency, dominant hand or university affiliation, and randomly assigned to 2D (*n* = 22) and 3D (*n* = 23) groups. Only the participants with a normal or corrected-to-normal vision were selected. All the participants completed the tasks using 2D and 3D monitors at the same setting and on the same day.

For this investigation, the Karl Storz SZABO-BERCI-SACKIER laparoscopic box trainer was used, holding a 10-mm camera port and two 5-mm working ports in a triangle position. The technical specifications of the applied imaging system were as follows: 3D video endoscope IMAGE 1 S 3D with TIPCAM 1 S 3D LAP (10-mm diameter, 30° optics); connect module: IMAGE1 S CONNECT and IMAGE1 S 3D-LINK (Karl Storz SE & Co. KG, Tuttlingen, Germany); 32” 3D monitor EJ-MDA32 (Panasonic Canada Inc., Ontario, Canada). The mode change from 2D to 3D was done at the video endoscope.

### Performance Task

**Figure 1:**
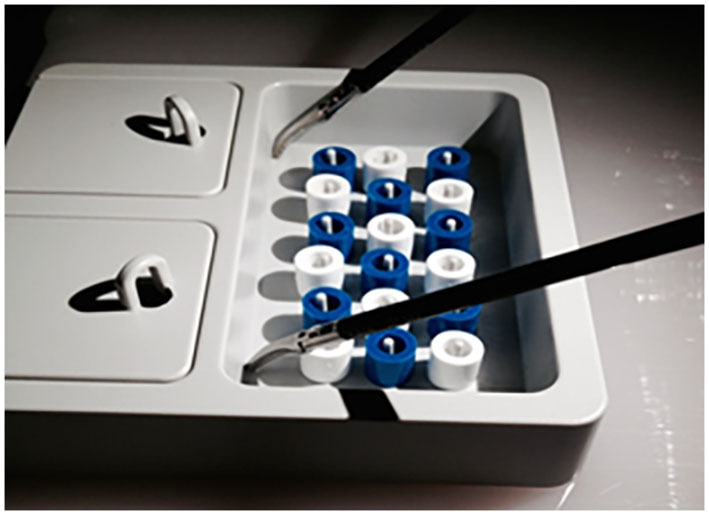
A practice module “Pack your luggage” of the Luebeck Toolbox: Open-box and sorting sleeves according to color.

For our investigations, the standard task “peg transfer” of the laparoscopy boxtrainer “LuebeckToolbox” (6) was used, in which white and blue sleeves in mixed positions have to be sorted according to color in two boxes with a hinged lid. In the beginning, all instruments are placed in the upper left and right corners. Time measurement was started, and the first sleeve was grasped with the instrument in the dominant hand. After opening the lid of the diagonally opposite box with the non-dominant hand, sleeves had to be transferred into the box, followed by closing the lid again. The next sleeve was transferred into the other box with the non-dominant hand in the same manner. Lost sleeves had to be picked up again, and all lids had to be closed before the next sleeve could be transferred. After all sleeves had been color sorted appropriately into the boxes, instruments were brought back into the neutral position (Figure 1). Time measurement was stopped and an error log was created. To determine the learning curve, the exercise was carried out 10 times. Performance time and the error rate of the 10 trials were recorded.

### Statistical Analysis

Baseline characteristics between participants of the two experimental groups were compared using the Fishers exact test for categorical variables and independent samples *t*-test for age. Normality distribution assumption was checked graphically and by Shapiro–Wilk test for performance times stratified by the laparoscopic group and trial.

Primary endpoints were operation times, and error rates were deemed as secondary endpoints. To analyze operation times, a two-way repeated measurements ANOVA with the effect of trail (that is the repeated measurement factor) and the main effect of laparoscopic condition (2D vs. 3D) was performed. Furthermore, the interaction between condition and trial was entered to assess whether learning curves differ between conditions. Differences in operating times between laparoscopic conditions would result in a significant main effect of that factor. If the participants showed a steeper learning curve in one laparoscopic condition, this would result in a significant interaction effect between time and condition.

*Post-hoc* tests for the repeated measurement factor were performed using pairwise dependent *t*-tests with Bonferroni–Holm adjustment to control for alpha-failure inflation due to multiple comparisons. In a sensitivity analysis, *post-hoc* comparisons were additionally stratified by laparoscopic condition.

Error rates were described descriptively and compared between laparoscopic conditions by the Mann–Whitney test. To take longitudinal data structure and discrete nature of error counts into account, a generalized linear mixed model (GLMM) with negative binomial residual distribution (due to substantial overdispersion) and log-link was performed. The main effects of laparoscopic condition and repeated measurement and interaction between both were entered as predictors. Model performance was assessed by Akaikes Information Criterion (AIC), root-mean-squared error (RMSA), and pseudo-R^2^ (Nagelkerke). A hypothesis for effects on error rates was analogous with operation times. Trial effects in the GLMM were reported by exponentiated model coefficients and their 95% confidence intervals. For the trial factor, the first trial was set as the reference category. For a graphical presentation of error rates, displaying mean or median values is inadequate, and would result in substantial loss of information. Therefore, failure rates in each trial were depicted by density plots (also known as violin plots), stratified by laparoscopic condition. Solid and dashed lines in the plot represent median and lower/upper quartiles, respectively. The mean error rate in each trial is depicted by the black dot within the violin.

According to the training instructions (http://www.luebeck-toolbox.com/training.html), two types of errors were recorded: dropping the sleeve between grasping and placement in the box (drop sleeve errors) and incomplete closure of the box (open box errors). The number of errors was compared for each type between the two laparoscopic modes using the Mann–Whitney test.

Data were stored in Microsoft Excel, and analyses were performed with R (version 4.1.1, R Foundation for Statistical Computing, Vienna). Values of *p* < 0.05 were considered statistically significant.

### Statement of Ethics

Written informed consent was obtained from those who agreed to participate. The article is excempted from ethical committee approval since that has not been necessary according to recommendation of the DFG (German association of research—“Deutsche Forschungsgesellschaft”). Neither there have been any risks during the performance task nor any unclear examinations or operations at the patients.

## Results

**Figure 2:**
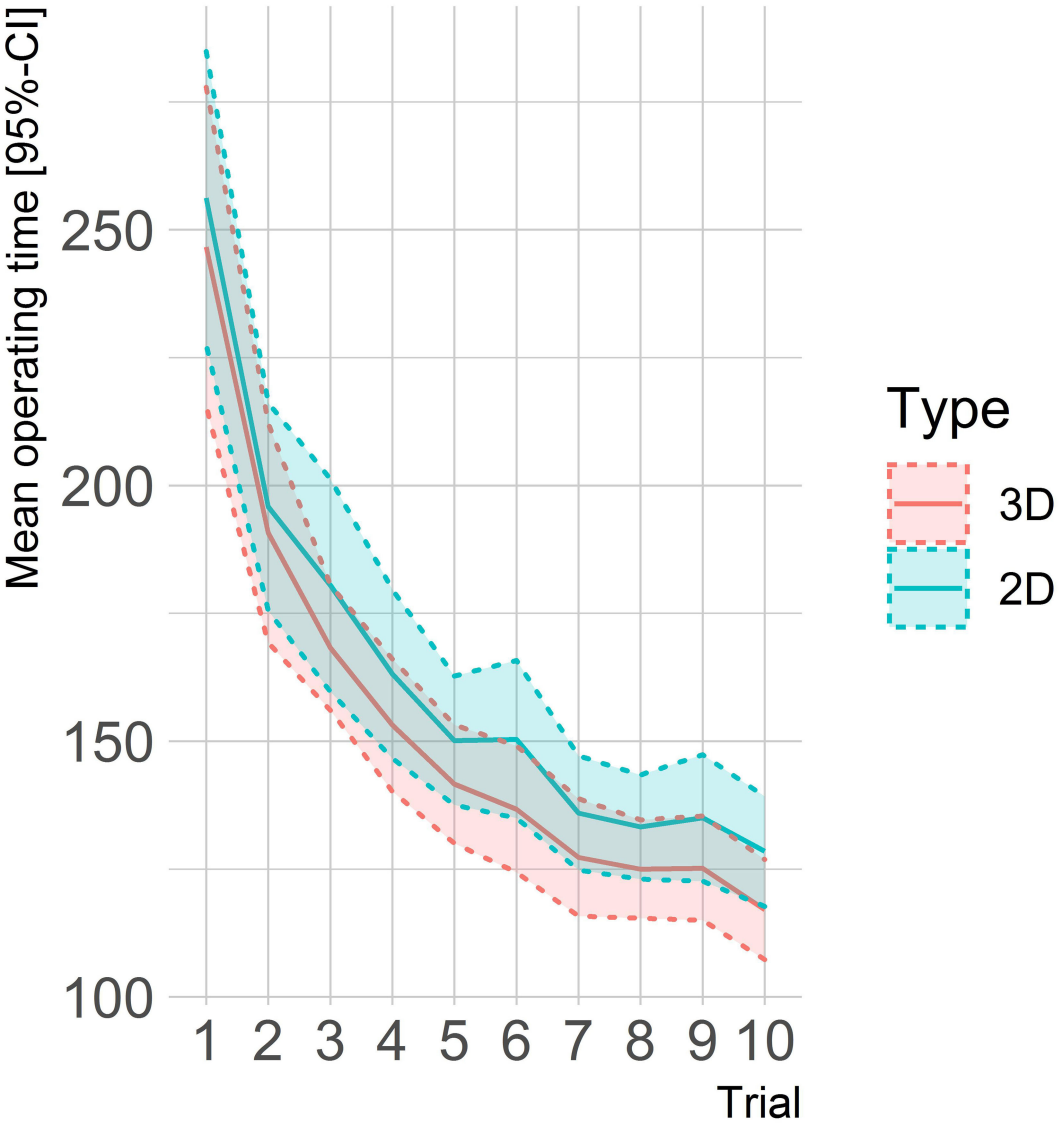
Mean operating time and 95% confidence bands.

The age range of the participants was 18–35 years, with a mean age of 24. ± 3.3 years in the 2D group and 23.4 ± 2.9 years in the 3D group (*p* = 0.83). Both groups did not significantly differ with respect to gender, wearing of glasses, frequency of video gaming, dominant hand, and university affiliation (Table 1). The mean values of time required to perform each of the 10 test runs showed no significant difference between the 2D/HD and 3D groups (Figure 2).

**Table 1:** Background of the participants.

	**2D group** **(*n =* 22)**	**3D group** **(*n =* 23)**	***P*-value**
Gender (male/female)	14:8	15:8	0.912
Right-/left-hander	21:1	20:3	0.608
Spectacle wearer (yes/no)	10:12	11:12	0.873
Prospective career in medicine/technology/other	9:6:7	9:8:6	0.963
Active video gamer Regular/past/no	7:5:10	11:3:9	0.492

### Operating Times

Operation times were reasonably normally distributed; however, the Mauchly test revealed a violation of the sphericity assumption (*p* < 0.001) so that a Greenhouse–Geisser correction was applied (ε_GG_ = 0.428). The ANOVA showed a highly significant and strong effect of time [*F*_(3.85, 161.7)_ = 155.9, *p* < 0.001, $\eta_{\text{g}}^{2}$ = 0.554]. With mean operating times of 251 s (SD = 67.9 s) in the first trial dropping to 123 s (SD = 23.9 s) in the 10th trial. The between-subject main effect of the laparoscopic condition failed to reach significance [*F*_(1,42)_ = 2.79, *p* = 0.10, $\eta_{\text{g}}^{2}$ = 0.042]. However, descriptively comparing operation times between laparoscopic conditions at each single trial showed slightly shorter operation times in the 3D condition for each and every comparison (see Figure 2). The interaction between laparoscopic condition and trial was not significant all [*F*_(3.85, 161.7)_ =0.342, *p* = 0.84, $\eta_{\text{g}}^{2}$ = 0.003].

As the main effect of laparoscopic condition was not significant, *post-hoc* tests of the trial effect on operation time were assessed in a pooled analysis. Adjusted pairwise *t*-tests showed no substantial time improvements from the 7th trial onward (*p*_adjusted_ > 0.11, with the exception of a significant difference between 7th and 10th trials, *p*_adjusted_ = 0.004). Additionally, we performed the *post-hoc* tests stratified by laparoscopic condition, mainly resulting in the same time effects as in the pooled analysis.

### Error Rates

**Figure 3:**
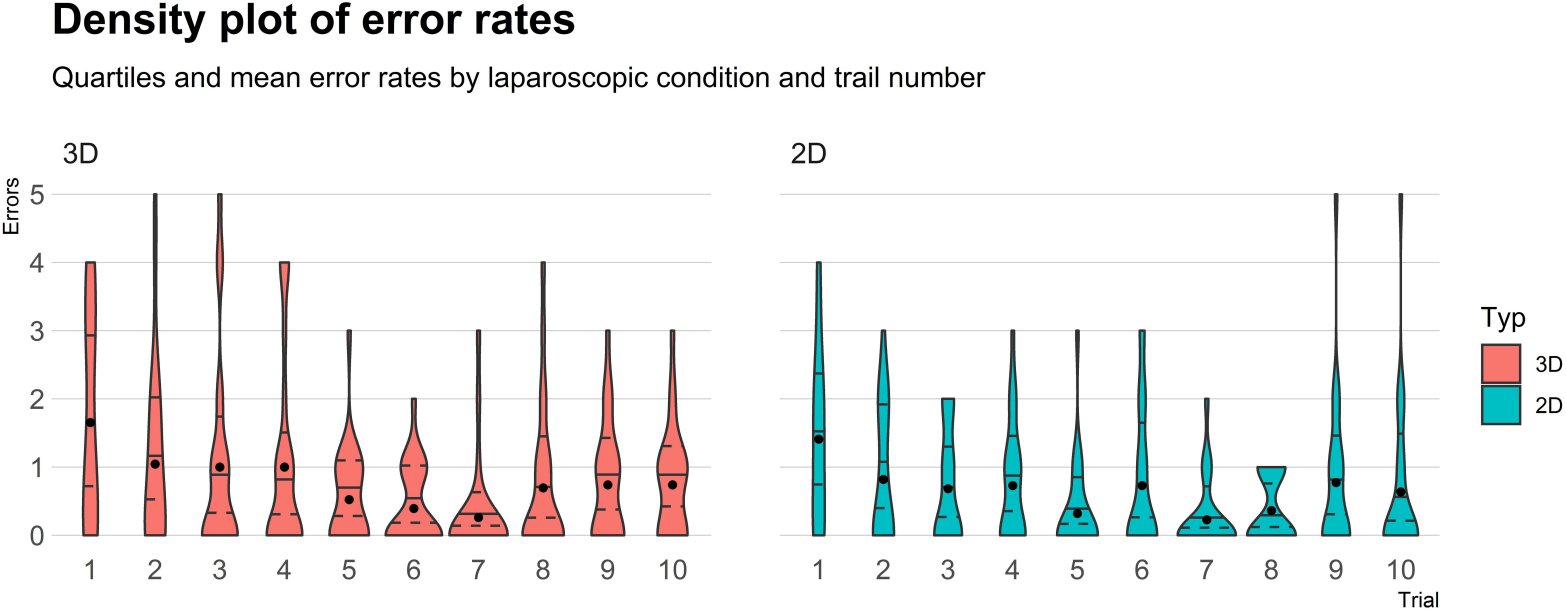
A density plot of error rates.

Distribution of errors (see Figure 3), within each trial and across all trials, was heavily right-skewed. Within each trial, the number of errors ranges between 0 and 5, with a median of 0 errors (IQR: 0–1). The overall number of errors, summarized across all trials, ranges between 1 and 24, with a median of 6 (IQR: 3–6). Stratified by laparoscopic condition, the participants showed a median of 5.5 errors (IQR: 3.3–7.8) in the 2D condition and 6. errors (IQR: 3–12.5) in the 3D condition, indicating no significant differences between groups (*p*_MW−Test_ = 0.72, *d* = 0.11).

The GLMM (*AIC* = 1,059, *RMSE* = 1.02, *R*^2^_Nagelkerke_ = 0.17) only showed a significant time effect (*p* < 0.001). Neither laparoscopic condition (β = −0.15, *SE* = 0.42, *p* = 0.72) nor the interaction between condition and trial (*p* = 0.83) was significantly associated with error rates. A significant error reduction (compared to the first trial) was observed from the 5th trial onward [*b*_5.*trial*_ = 0.32, 95%-CI: (0.14, 0.67), with a minimum failure rate in the 7th trial (*b*_7.trial_ = 0.16, 95%-CI: (0.06, 0.39)].

**Figure 4:**
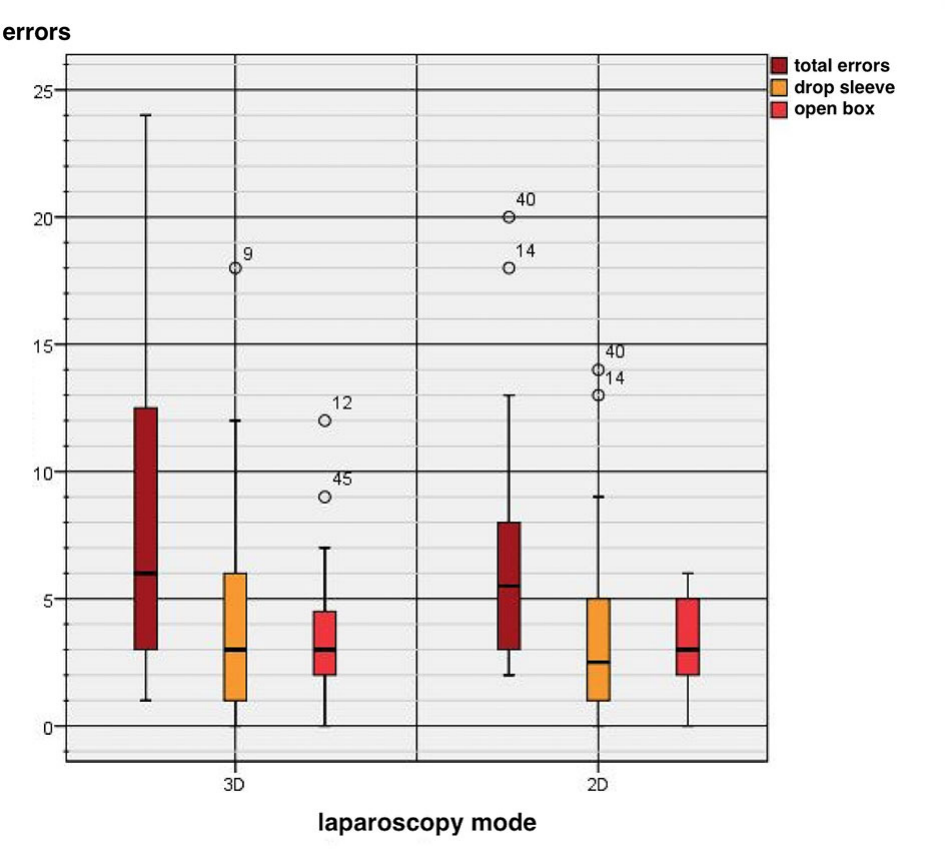
Comparison between two-dimensional (2D) and three-dimensional (3D) systems regarding technical errors.

Two types of errors (drop sleeve errors and open box errors) were recorded. The error rate of the 10 test runs is depicted in Figure 4. Because error types did not differ significantly (*p* = 0.0715) between the laparoscopic modes, the errors were subsequently analyzed together.

## Discussion

The current state-of-the art operating theaters are 3D/HD, 2D/HD, and 2D/4K systems, whereby 4K resolution monitors introduced a few years ago definitely brought about an improvement of the visual orientation at the operation site (7). Many studies in the past comparing the surgical performance of these different visualization systems, however, yielded quite heterogeneous results, which are, apparently, also dependent on the laparoscopic tasks to be performed and/or the skills of the respective surgeon.

A systematic review by Sørensen et al. in 2016 assessing 31 randomized studies demonstrated a certain advantage of 3D laparoscopy over 2D/HD in primarily simulated settings (3). The operating time under 3D vision was significantly reduced in 71% of the randomized controlled trials, the error rate is 63%. A systematic review of laparoscopic cholecystectomy by Komaei et al. showed a significant advantage of 3D laparoscopy in 60% with respect to operating time (8), while two recent clinical studies comparing 2D/HD vs. 3D laparoscopic right hemicolectomy detected no significant difference with respect to intra- and postoperative complications and confirm equivalent patient outcomes (9, 10). When interpreting the results for the different technologies, many factors besides the technological improvements over the years have been taken into account, e.g., if the participants in these studies were laparoscopic novices or experienced surgeons. Harada et al. reported that expert laparoscopic surgeons, despite very good experiences with 3D/HD systems, still see an advantage in the 2D/4K technology for tasks in narrow spaces (7).

Our study was mainly aimed to assess and to question previous study findings as an essential part of the research in this field. The replication of data increases the acceptance of previous studies but also promotes critical discussion as a part of a modern error culture. The common goal is the optimal training of young surgeons. Which practical implementations should we draw to provide an efficient clinical training for future surgeons inexperienced in laparoscopic techniques? And are the frequently stated advantages of 3D technologies so convincing that 2D technologies should not be used in the future, even though this would require a complete and costly exchange of the clinical equipment? To answer these questions, our study was accordingly limited to laparoscopic novices in a standardized box trainer setting.

The Luebeck Toolbox is an established training tool for basic minimally invasive surgery skills (11). The participants were asked to perform a simple test, the “peg transfer” of the “Luebeck Toolbox” in 10 replicates. Measurements were operating time and number of errors, both target criteria in the comparison of 3D and 2D laparoscopy for everyday clinical practice. Mean values of test times did not significantly differ between 2D/HD and 3D groups. In the first test runs, a similar learning curve with significance (*p* < 0.05) was demonstrable for both groups. From the 5th attempt in the 2D group and from the 6th attempt in the 3D group onward, no significant difference could be detected anymore. In pairwise comparisons, the operating time was no longer significantly reduced after the 7th attempt. With respect to the error rate, no significant difference between the 2D and 3D groups was observed.

Two types of error (drop sleeve errors and open box errors) did not differ significantly between the laparoscopic modes.

Our results are, partially, in contrast to other studies, thus confirming the divergence of current studies, comparing the benefits of 3D vs. 2D techniques with respect to a reduction of performance time and better performance. Poudel et al. demonstrated in a similar investigation with 44 students per group a significant advantage of the 3D group in operation time and the error rate (12). A comparable result was obtained in a study with 50 novices by Schoenthaler et al. (13). Despite the dominance of 3D laparoscopy in many studies, one-third of the studies found no significant differences in 2D applications, and, apparently, many medical students experience difficulties when switching to 2D devices after having been trained in 3D environments, which is reflected by poorer performance (12, 14). Thomaschewski et al. reported comparable learning curves in confined spaces for 3D and 2D/4K resolution (15), indicating that both systems are equally suited.

The findings of this study demonstrate that laparoscopy novices perform simple tasks without any differences between 2D/HD and 3D techniques concerning learning speed and the error rate. We currently see no need to exchange existing 2D equipment in clinics for training purposes, especially if an upgrade to 2D/4K resolution is possible. For more challenging tasks in simulated settings or the improvement of surgical performance in daily clinical practice (which was not assessed here), 3D systems may yield better results than 2D/HD systems.

### Strengths and Limitations of the Study

Our study has a number of important strengths. The study participants were medical students who were prepared for practical surgical activities with this exercise according to their study progress. Therefore, the validity of the generalization of the results to other medical students with this level of training can be assumed. The clear and standardized execution of the experiment by means of the scientifically evaluated Luebeck toolbox provides a high degree of objectivity, validity, and comparability. The replication of data increases the acceptance of previous studies, but also promotes critical discussion as a part of a modern error culture.

Finally, our study was a non-industry-funded trial. Our study and the results have scientific integrity and independence. Probst et al. show that studies with industry funding lead to exaggerated positive reporting of outcomes. They reported in the analysis of 165 randomized controlled trials about a positive outcome in 76.5% of industry-funded trials and in 38.% of non-industry-funded trials (16).

The following are limitations of the study. It is unclear whether results of our study using Luebeck toolbox are transferable to the operation room. Our students were novices with no experience in laparoscopic surgery. It is questionable whether our results are transferable to experienced surgeons.

Our study design included 10 trials to record the performance time and the error rate. We performed a sample size calculation and endpoints. In our pilot study using the 2D technique, test persons (*n* = 3) started, on average, with 224 s in Experiment 1 and ended, on average, at 152 s in Experiment 10.

We defined a 15% reduction in time (23 s) for 3D compared to 2D technique as a meaningful improvement. With an estimated standard deviation of 26 s in the pilot study, a group size of 22 test persons is required to achieve a power of 80% by assuming the usual alpha level of 5%. Laubert et al. reported a median of approximately 32 repetitions to reach expert performance (experienced surgeons with a least 500 minimally invasive surgeries) of 72 s. Therefore, it cannot be ruled out that continuing the task might potentially result in a significant difference in later trials. However, the most important learning curve differences were reported in the early trials (11).

## Conclusions

The results of our study with laparoscopically inexperienced students revealed no significant differences with respect to performance time and the error rate between 2D/HD and 3D technology for a simple standardized task. Both techniques are thus equally suited for the training of future surgeons, and we see no need to exchange existing 2D systems in clinics. With its critical analysis, the study provides a knowledge gain on this topic, supports a differentiated view, and reflects the daily praxis in German clinics where both technologies successfully exist in parallel.

## Data Availability Statement

The original contributions presented in the study are included in the article/supplementary material, further inquiries can be directed to the corresponding author/s.

## Ethics Statement

Ethical review and approval was not required for the study on human participants in accordance with the local legislation and institutional requirements. The patients/participants provided their written informed consent to participate in this study.

## Author Contributions

MS, CD, AG, CH, and RM contributed to conception or design of the work, data analysis and interpretation, critically revised the article, and finally approved the version to be published. CD, AG, and CH contributed to data collection. MS, CD, and RM drafted the article. All authors contributed to the article and approved the submitted version.

## Conflict of Interest

The authors declare that the research was conducted in the absence of any commercial or financial relationships that could be construed as a potential conflict of interest.

## Publisher's Note

All claims expressed in this article are solely those of the authors and do not necessarily represent those of their affiliated organizations, or those of the publisher, the editors and the reviewers. Any product that may be evaluated in this article, or claim that may be made by its manufacturer, is not guaranteed or endorsed by the publisher.

## Abbreviations

AIC: Akaikes Information Criterion
2D: two dimensional
3D: three dimensional
DFG: German association of research
GLMM: generalized linear mixed model
HD: high definition
RMSA: root-mean squared error.
